# Roles of bulk and surface magnetic anisotropy on the longitudinal spin Seebeck effect of Pt/YIG

**DOI:** 10.1038/s41598-017-13689-2

**Published:** 2017-10-17

**Authors:** Vijaysankar Kalappattil, Raja Das, Manh-Huong Phan, Hariharan Srikanth

**Affiliations:** Department of Physics, University of South Florida, Tampa, FL 33620 USA

## Abstract

A clear understanding of the temperature evolution of the longitudinal spin Seebeck effect (LSSE) in the classic Pt/yttrium iron garnet (YIG) system and its association with magnetic anisotropy is essential towards optimization of its spin-caloric functionality for spintronics applications. We report here for the first time the temperature dependences of LSSE voltage (*V*_LSSE_), magnetocrystalline anisotropy field (*H*_K_) and surface perpendicular magnetic anisotropy field (*H*_KS_) in the same Pt/YIG system. We show that on lowering temperature, the sharp drop in *V*_LSSE_ and the sudden increases in *H*_K_ and *H*_KS_ at ~175 K are associated with the spin reorientation due to single ion anisotropy of Fe^2+^ ions. The *V*_LSSE_ peak at ~75 K is attributed to the *H*_KS_ and *M*_S_ (saturation magnetization) whose peaks also occur at the same temperature. The effects of surface and bulk magnetic anisotropies are corroborated with those of thermally excited magnon number and magnon propagation length to satisfactorily explain the temperature dependence of LSSE in the Pt/YIG system. Our study also emphasizes the important roles of bulk and surface anisotropies in the LSSE in YIG and paves a new pathway for developing novel spin-caloric materials.

## Introduction

Spin caloritronics based on the spin-Seebeck effect (SSE) is an emerging area of research owing to its potential use in advanced spintronics devices^1,2^. SSE is associated with the generation of pure spin current without charge current when a thermal gradient is established in the presence of an applied magnetic field^3^. Since the discovery of SSE in a ferromagnetic metal (NiFe), it has been reported in a wide range of materials, including ferromagnetic insulators, ferromagnetic semiconductors, and non-magnetic materials, using longitudinal and transverse measurement configurations (known as LSSE and TSSE, respectively)^3–7^. The great diversity of host materials raises an important question about the underlying physical origin of the SSE^8–11^. In particular, the origin of the temperature dependence of the LSSE in the Pt/YIG system has been a subject of long lasting debate^9^. Siegal *et al*. observed a decrease in the LSSE voltage (*V*_LSSE_) with decreasing temperature in Bi-YIG thin films, with a sudden change of *V*_LSSE_ slope below 200 K^11^. While the origin of the sudden change in *V*_LSSE_ below 200 K was unclear^11^, this may be associated with spin reorientation due to single ion anisotropy of Fe^2+^ ions that was also reported to occur below 200 K in the single crystalline YIG^12^.

It has recently been reported that when the thickness of YIG exceeds its magnetic domain size (~5 μm), the magnetic field-dependent *V*_LSSE_ shows an anomaly in a low field regime (<±0.3 kOe)^13^. This low field feature has been attributed to the presence of an intrinsic easy axis perpendicular magnetic anisotropy (PMA) at the YIG surface^13^. The presence of PMA has been experimentally shown at 300 K to influence the value of the saturation magnetic field $({H}_{Sat}^{LSSE})$ of a saturated *V*_LSSE_^14,15^. These experimental observations indicate the possible role of PMA in the LSSE in YIG.

Furthermore, a maximum in *V*_LSSE_ around 75 K has been reported in both bulk and thin films of YIG^9^. While this enhancement was explained by the magnon-phonon drag model^8,16^, a recent study on the temperature dependences of thermal conductivity (*σ*) and *V*_LSSE_ of YIG has revealed a maximum in *σ*(*T*) around 25 K^17^, which is about 50 K below the *V*_LSSE_(*T*) peak (~75 K)^8,9^, thus questioning about the validity of the existing magnon-phonon drag model. Most recently, the temperature dependent LSSE with a peak around 75 K has been reasonably well explained using the magnon-mediated model that considers the temperature-dependent effective propagation length of thermally excited magnons in bulk YIG^9^. This study also highlights the important role of interface effects. However, the emerging question about the effect of the PMA on the LSSE remains unaddressed.

To shed some light on these important issues, we have simultaneously studied the temperature dependence of the effective magnetic anisotropy and *V*_LSSE_ in the same single crystal of YIG, using the radio-frequency transverse susceptibility (TS)^18^ and LSSE techniques, respectively. Over the years, our group has validated TS as a direct probe of effective magnetic anisotropy in a large class of magnetic materials ranging from thin films^19^, single crystals^20^ to nanoparticles^21^. In particular, we have used this technique to unravel the unusual magnetic behavior often seen in manganites^22^ and to probe the magnetocrystalline anisotropy-driven phase transition in cobaltites^23^. In this study, the bulk magnetocrystalline anisotropy field (*H*_K_), the surface PMA field (*H*_KS_) and their temperature evolutions of YIG are probed using the TS technique. Coupled with the temperature evolution of V_LSSE_, we show that on lowering temperature, a sudden decrease in *V*_LSSE_ at ~175 K corresponds to the sudden increases in *H*_K_ and *H*_KS_, arising from the spin reorientation that occurs at the same temperature. At lower temperatures (*T* < 125 K), *V*_LSSE_(*T*) shows a peak at ~75 K which is associated with the *H*_KS_ and *M*_S_ (saturation magnetization) whose peaks also occur at the same temperature.

## Results and Discussion

**Figure 1:**
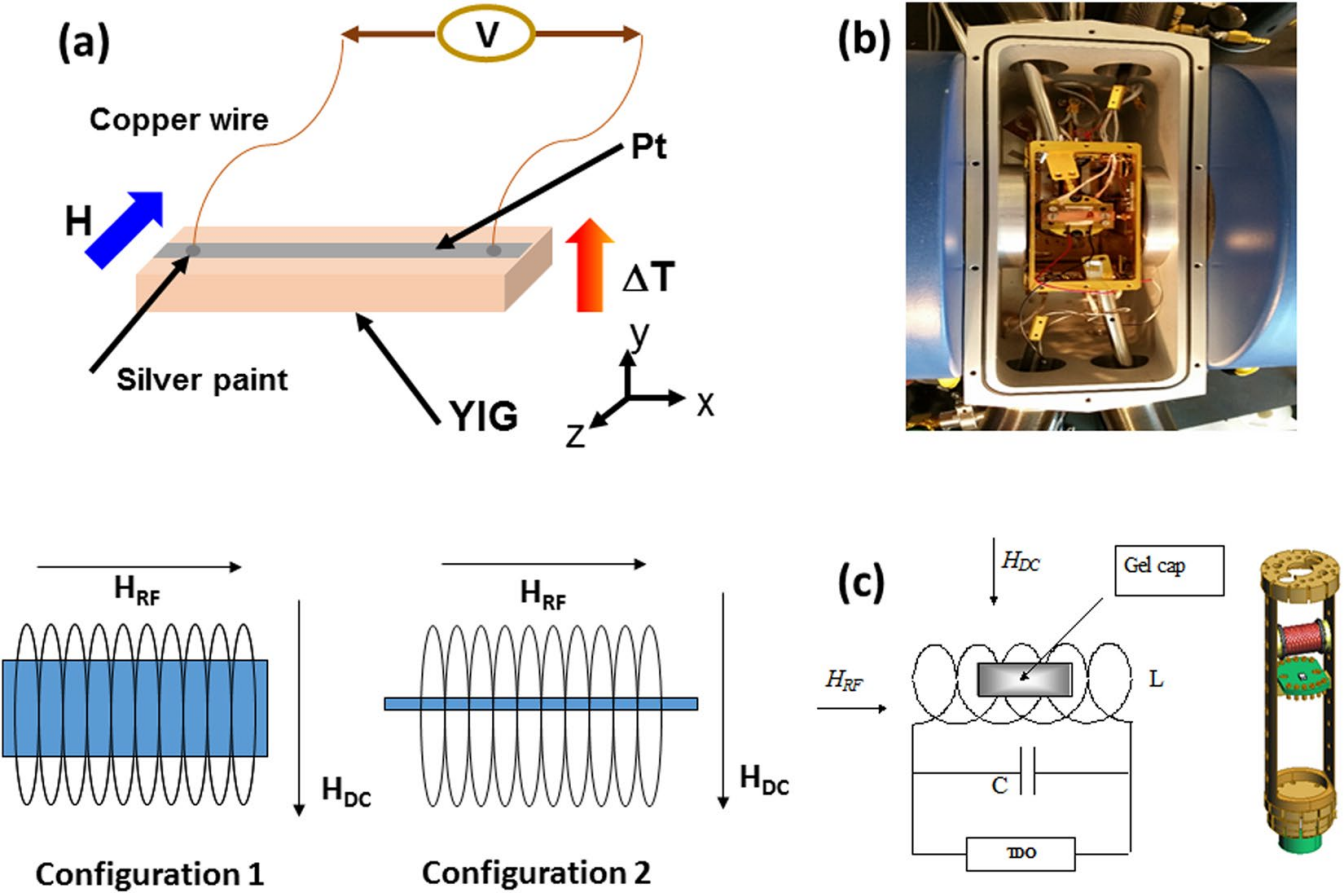
(**a**) A schematic illustration of the longitudinal Spin Seebeck effect (LSSE) measurement set-up; (**b**) a picture of the experimental set-up developed to measure LSSE; (**c**) schematic illustration of the transverse susceptibility measurement set-up and measurement configurations (configuration 1: in-plan TS experiment; configuration 2: out-of-plane TS experiment).

The same sample (single crystal: Y_3_Fe_5_O_12_ or YIG) was used for LSSE and TS measurements. Details of these experiments are presented in the Methods section. Figure 1 shows schematic illustrations of the LSSE and TS setups, with which the temperature dependences of *V*_LSSE_, *H*_K_ and *H*_KS_ have been studied in detail.

**Figure 2:**
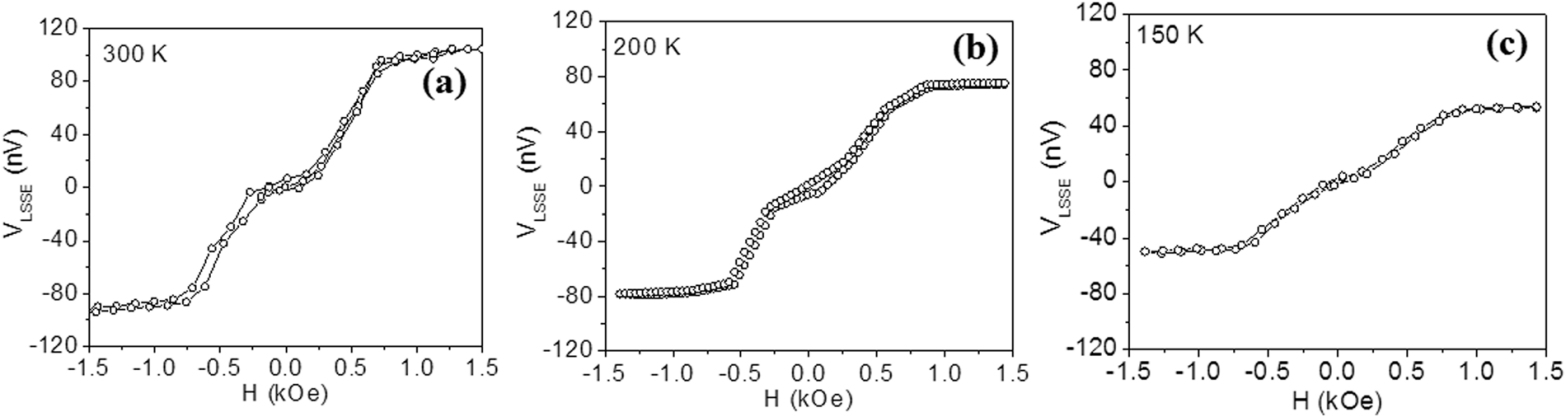
Magnetic field (*H*) dependence of the longitudinal spin Seebeck voltage (*V*_LSSE_) in the Pt/YIG sample at Δ*T* = 2 K measured at (**a**) 300 K, (**b**) 200 K, and (**c**) 150 K.

In the following section, we present and discuss the LSSE results. Figure 2 shows the magnetic field dependence of *V*_LSSE_ of Pt/YIG for a temperature gradient of Δ*T = *2 K at representative temperatures of 300, 200, and 150 K. It is observed that in the low field region (*H* ≤ ±0.3 kOe), *V*_LSSE_ is relatively small (almost zero) and remains almost unchanged with increasing magnetic field. This behavior was also reported in literature for YIG when the sample thickness exceeded its magnetic domain size (~5 μm)^13^. The *V*_LSSE_ increases rapidly in the field range ±0.3 kOe < *H* < ±0.7 kOe and becomes saturated for *H* ≥ ±0.7 kOe. The low magnetic field behavior of the YIG/Pt structure was observed at all measured temperatures. While the magnitude of *V*_LSSE_ decreases with lowering temperature, the critical field (*H*_cri_ = ±0.3 kOe) for the *V*_LSSE_ increase remains almost unchanged.

**Figure 3:**
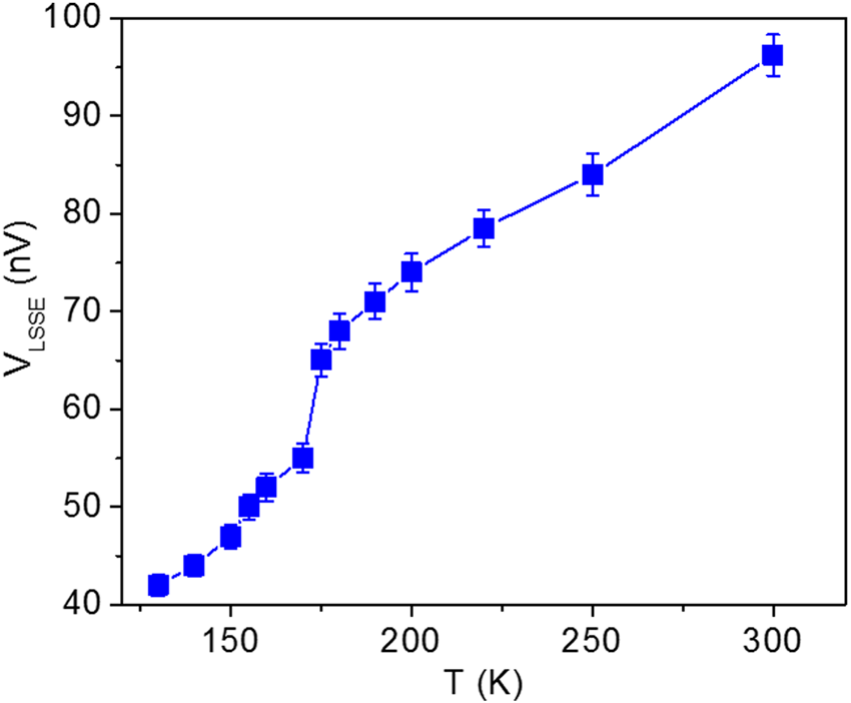
Temperature dependence of saturated LSSE voltage for the Pt/YIG sample.

Figure 3 shows the temperature dependence of the saturated *V*_LSSE_ for a temperature gradient of Δ*T* = 2 K. The saturated value of *V*_LSSE_ is defined as *V*_LSSE_ = ∆*V*/2, where ∆*V* is the difference between maximum values of the positive and negative saturation. It can be observed in Fig. 3 that *V*_LSSE_ decreases with lowering temperature in the investigated temperature range (125 K–300 K). This trend was also observed by Siegel *et al*. for a Bi-doped YIG thin film^11^. The *V*_LSSE_ at 300 K is determined to be ~96 nV, which drops to 47 nV at 150 K. The obtained values of *V*_LSSE_ are in the expected range reported in literature^8^. It is worth noting in Fig. 3 that there is a sudden drop in *V*_LSSE_ just below ~175 K, and this feature was also observed for the Bi-YIG thin films, although its origin was not understood^11^. While this drop in *V*_LSSE_ cannot be explained by the theoretical models proposed for the SSE, we recall that the exchange energy associated with the first body mode (spin wave with the smallest real wave number) varies with changes in magnetization and magnetocrystalline anisotropy^24^. The temperature dependence of the photo-induced shift of the surface modes relative to the first body mode of YIG single crystals has shown a negligible value above 175 K^24^, and a Mössbauer study has revealed a spin reorientation below 175 K due to the single ion anisotropy of Fe^+^^2^ ions^12^. These observations suggest that the sudden decrease in *V*_LSSE_ below ~175 K, as observed in our study and also reported in the literature^11^, is likely associated with the spin reorientation that occurs at the same temperature. When spin reorientation happens in a material, its preferred magnetization direction changes which, in turn, alters the effective magnetic anisotropy and the LSSE. This behavior is similar to the large changes in V_LSSE_ observed in antiferromagnetic systems due to spin-flop transitions^25^. Since LSSE experiments were performed on bulk YIG, the effects of spin reorientation and magnetic anisotropy on the LSSE in orthogonal orientations with respect to the applied magnetic field would be significant.

In order to elucidate these intriguing features, we have studied the temperature evolution of the magnetic anisotropy of YIG in both in-plane and out-of-plane directions by radio-frequency transverse susceptibility (TS), using a self-resonant tunnel diode oscillator with a resonant frequency of 12 MHz and sensitivity of the order of 10 Hz^18^. In the TS method, as the DC field is swept from positive saturation to negative saturation of the sample under study, the resonant frequency of the coil with the sample changes proportional to the transverse susceptibility of the sample as follows:

$$\frac{{\rm{\Delta }}{\chi }_{T}}{{\chi }_{T}}({\rm{ \% }})=\frac{|{\chi }_{T}(H)-{\chi }_{T}^{Sat}|}{{\chi }_{T}^{Sat}}\times 100$$

where ${\chi }_{T}^{{Sat}}$ transverse susceptibility at a saturation field *H*_sat_. It has been theoretically shown that a ferromagnetic material should yield TS peaks at the anisotropy fields (±*H*_k_) and switching fields (−*H*_S_) as the DC field is swept from positive to negative saturation and vice versa^26,27^. However, in some cases where *H*_K_ values are very close to *H*_S_, the switching peak is often merged with one of the anisotropy peaks in a unipolar scan of the field for example from to positive to negative saturation.

**Figure 4:**
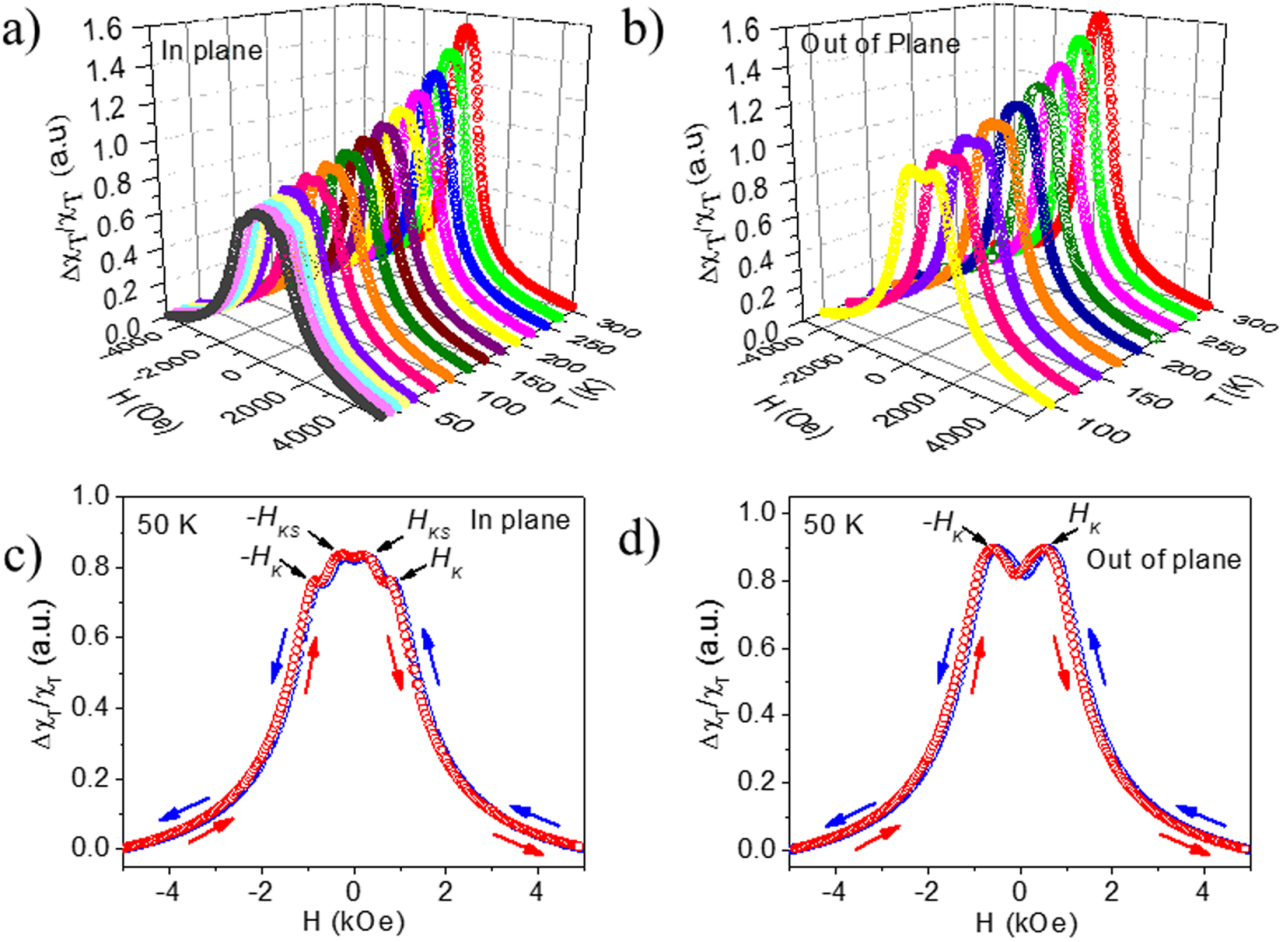
Bipolar transverse susceptibility (TS) scans taken at different temperatures for the single crystal YIG in (**a**) in-plane and (**b**) out-of-plane directions; (**c,d**) bipolar TS scans for the same at 50 K.

**Figure 5:**
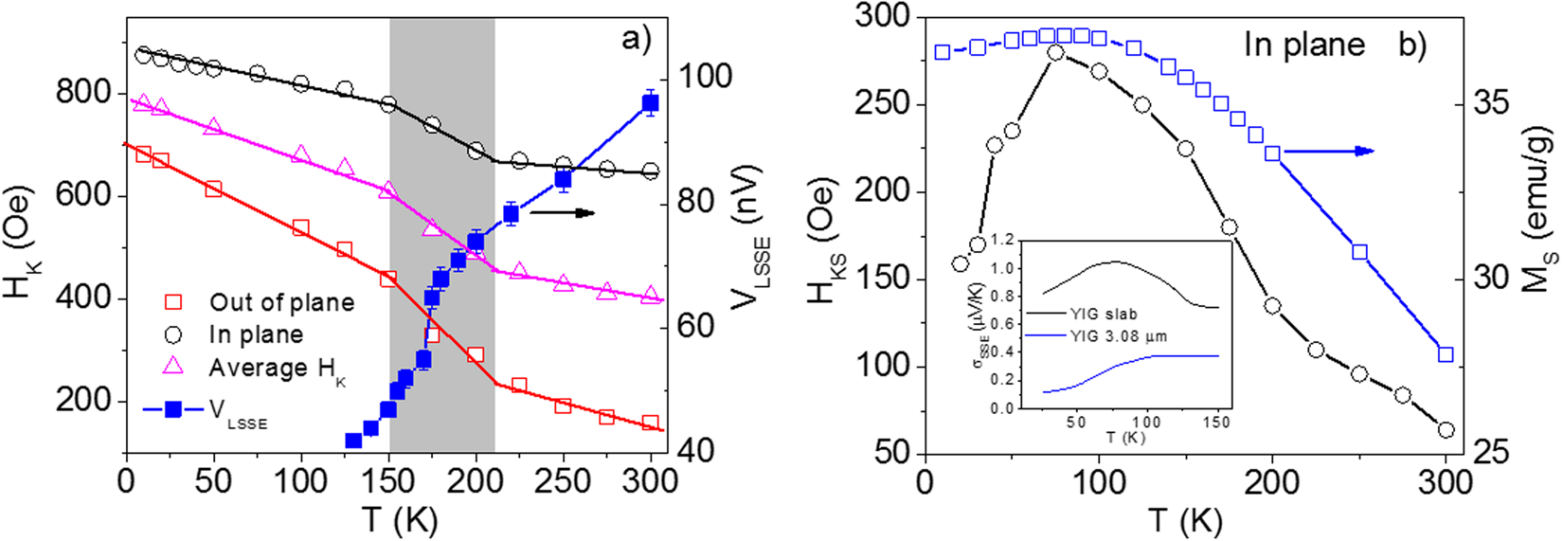
Temperature dependences of (**a**) *H*_K_ in the out-of-plane and in-plane directions and their subtraction, and the saturated LSSE voltage measured with Δ*T* = 2 K; (**b**) M_S_ and *H*_K__S_ in the in-plane direction, with its inset showing the temperature dependence of LSSE voltage for both the single crystal and thin film of YIG as taken from ref.^9^.

Figure 4a and b show the 3D bipolar TS scans of YIG in the in-plane and out-of-plane directions in the temperature range of 20–300 K, respectively. It can be observed in Fig. 4a,c that as the DC magnetic field was swept from positive to negative saturation, in-plane TS scans showed two distinct peaks corresponding to ±*H*_K_ (the magnetocrystalline anisotropy field). Interestingly, two other anisotropic peaks corresponding to ±*H*_KS_ (the surface PMA field) also appeared to occur at lower DC fields, establishing different anisotropic behavior of the YIG surface. In the out-of-plane direction, switching and anisotropy fields are close to each other so that the observed peak appeared to merge together, on increasing the magnetic field from zero (Fig. 4b,d). As expected, no peaks associated with *H*_KS_ are observed in this measurement configuration as the applied magnetic field is parallel to the PMA direction. The temperature dependences of *H*_K_ for both the in-plane and out-of-plane directions and its normalized value are plotted in Fig. 5a. The temperature dependence of *H*_KS_ is also plotted in Fig. 5b. It can be seen in Fig. 5a that *H*_K_ shows an increase as the temperature is lowered from 300 K, which is expected for a typical ferromagnet. While the in-plane *H*_K_ increases from 670 Oe at 225 K to 876 Oe at 10 K, a much larger change in the out-of-plane *H*_K_ from 160 Oe at 300 K to 680 Oe at 10 K is observed. It is worth noting here that both the in-plane and out-of-plane anisotropy fields show a similar temperature dependence. For both the in-plane and out-of-plane directions there is a sharp increase in *H*_K_ at ~175 K, which corresponds to the sudden decrease in *V*_LSSE_, as clearly seen in the compiled plot of Fig. 5a. The temperature dependences of *H*_K_ (Fig. 5a) and *H*_KS_ (Fig. 5b) resemble that of *V*_LSSE_ in the investigated temperature range (125–300 K). We should note that while in the saturated state the magnetic anisotropy may not play a role for the magnetization configuration (which is aligned completely along the field), the magnonic spin current diffusion length that depends on the magnetic anisotropy likely plays a role^28,29^. Larger magnetic anisotropy might open a larger magnon gap, leading to only higher frequency magnons to propagate which have a shorter diffusion length^30^ and hence the smaller LSSE. Other effects such as magnon population change due to temperature could also impact the temperature dependence of LSSE^9^.

It is also worth noticing in Fig. 5a that *H*_K_ possesses a more dominant contribution from the out-of-plane component than from the in-plane component, providing the first direct experimental proof of a recent theoretical prediction^13^. The increase in *H*_K_ corresponds to the decrease in *V*_LSSE_, suggesting that LSSE should be exploited in systems with low magnetic anisotropy, and that LSSE can be tuned by manipulating the anisotropy of the material. We recall that the temperature dependences of the anomalous Nernst effect (ANE) and SSE studied in both the single crystal and thin film of Fe_3_O_4_ have also revealed strong decreases in the ANE and SSE voltages at temperatures below the Verwey transition temperature ~125 K, at which the material undergoes a cubic (metal) high-temperature to monoclinic (insulator) low-temperature transition, and below which the magnetocrystalline anisotropy is found to suddenly increase as well^31,32^. Due to the coexistence of both ANE and SSE in Fe_3_O_4_, however, it was not possible to decouple the SSE from the ANE, and consequently the temperature evolution of SSE could not be directly related to that of the magnetic anisotropy. Given the fact that the ANE effect is absent in YIG due to its insulating nature, and that YIG undergoes a spin orientation below 175 K due to the single ion anisotropy of Fe^2+^ ions, which, in effect, alters the magnetic anisotropy and hence the magnetization direction of the material, we can safely attribute the sudden change in *V*_LSSE_ to the intrinsic spin reorientation in YIG that affects the thermal spin injection in the FM/metal interface. These important findings indicate the possible coupling of anisotropy of the system to the LSSE voltage, which further validate the recent experimental and theoretical predictions that emphasize the role of magnetic anisotropy in SSE systems^11,13^.

Finally, we note in Fig. 5b the temperature dependence of *H*_KS_ in the low temperature region (*T* < 125 K). Clearly, *H*_KS_(*T*) shows a broad peak around 75 K, which corresponds to the broad peak of *V*_*L*SSE_(*T*) observed previously for bulk YIG (inset of Fig. 5b)^9^. While possible explanations have been put forward^9^ for the *V*_*L*SSE_(*T*) peak at ~75 K, no consensus has been formed about this. An important fact that emerges from our study is that the dependence of *V*_*L*SSE_(*T*) is related to the change in PMA (*H*_KS_), which is an intrinsic characteristic of YIG^15^. It has been theoretically shown by *Xiao et al*. that the presence of PMA at interface between YIG and Pt gives raise to spin wave excitation, thus increasing the excitation power^33^. Later *Uchida et al*. arrived at the same conclusion, through experimental and numerical calculations on YIG (both single crystal and film), that the difference between bulk magnetization and surface magnetization induced by PMA causes the low field anomaly of V_LSSE_ in the Pt/YIG system^13^. Micro-magneto optic Kerr effect measurements (MOKE) in a longitudinal configuration have mimicked the low field aberration similar to that observed in the LSSE measurements, proving the non-collinear alignment of spins between the bulk and surface of YIG^15^. Reproducibility of this low field anomaly independent of fabrication process shows it is rather an intrinsic property of YIG and attributed to the PMA. Our observation of *H*_KS_(*T*) at 75 K coincides with the *V*_LSSE_(*T*) peak (Fig. 5b and its inset), which suggests that the PMA has strong influence not only on the low field plateau but also on the saturated value of *V*_LSSE_. The sharp decrease in *H*_KS_ below ~75 K can be attributed to the rotation of surface spins away from the perpendicular easy-axis direction. This is possible in a magnetic system composed of two different surface and bulk spin configurations like YIG. Due to the different temperature response and alignment of the surface and bulk spins, there exists a temperature below which the surface spins are rotated away from their perpendicular direction by an internal magnetic field induced by the bulk spins, leading to a spin canting-like phenomenon. As a result, the total magnetization of YIG is reduced with lowering temperature below ~75 K, as shown in Fig. 5b. Our results can be corroborated with those reported by Guo *et al*.^9^, in order to explain the temperature dependence of *V*_LSSE_ with its anomalies at 175 K and 75 K in the Pt/YIG system. According to the magnonic spin current model^9^, with decreasing temperature the total number of magnons decreases while the effective thermal magnon propagation length increases. We find that the decrease in magnon number and the increase in magnetic anisotropy with temperature result in the decrease in *V*_LSSE_ in the high temperature region of 125–300 K, as contribution to the LSSE from the magnon propagation length is less dominant for *T* > 125 K^9^. The noted drop in *V*_LSSE_ at *T*~175 K, associated with the sharp increase in *H*_K_ and *H*_KS_, originate from the spin reorientation that occurs at the same temperature. With further decrease in the temperature (*T* < 125 K), however, the strong increase in the propagation length (also the strong increase in *M*_S_) contributes dominantly to the LSSE, leading to the increase in *V*_LSSE_ at 75 K < *T* < 125 K. At *T* < 75 K, the decrease in *V*_LSSE_ is attributed to the decrease in the total number of thermally excited magnons and *M*_S_ (Fig. 5b) and the increase in *H*_K_ (Fig. 5a). Since the magnon propagation length has been shown to be almost independent of temperature below 75 K^9^, the positive contribution to the LSSE from the decreased *H*_KS_ is less dominant in this temperature region. Nevertheless, further theoretical studies are needed to provide a microscopic quantitative understanding of the experimentally obtained results.

In summary, the temperature dependences of LSSE voltage and magnetic anisotropy of bulk YIG slabs have shown that the sharp drop in *V*_LSSE_ with respect to temperature at ~175 K is associated with change in magnetic anisotropy, which originates from the spin reorientation transition in YIG. The *V*_LSSE_ peak at ~75 K is also attributed to the surface PMA (*H*_KS_) and the *M*_S_ whose peaks also occur in the same temperature range. These effects of surface and bulk magnetic anisotropies are corroborated with those of thermally excited magnon number and magnon propagation length to explain the temperature dependence of LSSE in the Pt/YIG system. Our study also emphasizes the important role of magnetic anisotropy in the LSSE in YIG and validates the recent theoretical predictions about anisotropic SSE in magnetic materials thus providing a new pathway for developing novel spin-caloric materials through desired tuning of the magnetic anisotropy.

## Methods

### Sample characterization

Commercially available Yttrium Iron garnet (YIG) single crystals grown by the floating zone method along the (111) direction from Crystal Systems Corporation, Hokuto, Yamanashi, Japan were used for the present study.

### Measurements

Longitudinal spin Seebeck voltage measurements were performed on a YIG single crystal of dimension 6 mm × 3 mm × 1 mm (length × width × thickness). Platinum strip of 6 mm × 1 mm × 15 nm was deposited on YIG using DC sputtering. The sputtering chamber was evacuated to a base pressure of 5 × 10^−6^ Torr and Argon pressure of 7 mT during the deposition. DC current and voltage used for deposition were 50 mA and ~350 V, respectively. The schematic of the LSEE measurement set-up is shown in Fig. 1a. For LSSE measurements YIG/Pt was sandwiched between two copper plates. A Peltier module was attached to the bottom plate and top plate temperature was controlled through molybdenum screws attached to the cryogenic system. Temperature gradient of approximately 2 K was achieved by applying 3 A current to the Peltier module. K-type thermocouples were used to monitor the temperature of top and bottom plates. The picture of the experimental set-up is shown in Fig. 1b. After stepping the system temperature and Peltier module current, measurements were performed after 2 h of stabilization time. The SSE voltage was recorded as the magnetic field was swept between positive and negative saturation of YIG, using a Keithley 2182 Nano voltmeter.

Transverse susceptibility (TS) measurements were performed using a self-resonant tunnel diode oscillator with a resonant frequency of 12 MHz and sensitivity in resolving frequency shift on the order of 10 Hz^18^. The tunnel diode oscillator is integrated with an insert that plugs into a commercial Physical Properties Measurement System (PPMS, Quantum Design), which is used to apply dc magnetic fields (up to ± 7 T) as well as provide the measurement temperature range (10 K < T < 300 K). In the experiment, the sample is placed in an inductive coil, which is part of an ultrastable, self-resonant tunnel-diode oscillator in which a perturbing small RF field (*H*_AC_ ≈ 10 Oe) is applied perpendicular to the DC field. The coil with the sample is inserted into the PPMS chamber which can be varied the temperature from 10 K to 350 K in an applied field up to 7 T. A schematic illustration of the TS measurement set-up and measurement configurations is shown in Fig. 1c.
